# Experiment of Structural Geometric Morphology Monitoring for Bridges Using Holographic Visual Sensor

**DOI:** 10.3390/s20041187

**Published:** 2020-02-21

**Authors:** Shuai Shao, Zhixiang Zhou, Guojun Deng, Peng Du, Chuanyi Jian, Zhongru Yu

**Affiliations:** 1 School of Civil Engineering, Chongqing Jiaotong University, Chongqing 400074, China; 622150086086@mails.cqjtu.edu.cn (S.S.); guojunforsea@gmail.com (G.D.); dupeng_cqjtu@163.com (P.D.); chuanyi_jian_cqjtu@163.com (C.J.); zhongru_yu_cqjtu@163.com (Z.Y.); 2 College of Civil and Transportation Engineering, Shenzhen University, Shenzhen 518061, China

**Keywords:** structural geometry monitoring, computer-vision-based measurement technology, bridge safety, holographic visual sensor, dense full-field measurement, digital twins

## Abstract

To further improve the precision and efficiency of structural health monitoring technology and the theory of large-scale structures, full-field non-contact structural geometry morphology monitoring is expected to be a breakthrough technology in structural safety state monitoring and digital twins, owing to its economic, credible, high frequency, and holographic advantages. This study validates a proposed holographic visual sensor and algorithms in a computer-vision-based full-field non-contact displacement and vibration measurement. Using an automatic camera patrol experimental device, original segmental dynamic and static video monitoring data of a model bridge under various damage/activities were collected. According to the temporal and spatial characteristics of the series data, the holographic geometric morphology tracking algorithm was introduced. Additionally, the feature points set of the structural holography geometry and the holography feature contours were established. Experimental results show that the holographic visual sensor and the proposed algorithms can extract an accurate holographic full-field displacement signal, and factually and sensitively accomplish vibration measurement, while accurately reflecting the real change in structural properties under various damage/action conditions. The proposed method can serve as a foundation for further research on digital twins for large-scale structures, structural condition assessment, and intelligent damage identification.

## 1. Introduction

Bridge engineering is not only the basis for traffic and transportation systems, but also an indispensable part of rapid progress in modern transportation. However, bridge structures suffer different degrees of damages and deterioration far before their designed lifetime due to material performance degradation and other influencing factors (e.g., environmental erosion, vehicle loads, wind loads, earthquakes and fatigue) in their entire service life. This may further affect transportation safety of bridge structures [1,2,3,4,5]. Concerning traditional structure management and maintenance detection methods, some defects can be found, including a high manual inspection cost, high subjectivity, high uncertainty, low efficiency, lack of scientifically quantified bases and failure in satisfying demands of engineering practice. In recent years, fast growth of relevant fields, such as modern sensing technology, control techniques, artificial intelligence, telecommunications, materials and data analysis, lays a technical and theoretical foundation for implementing structural health monitoring (SHM) technology in more direct and more cost-effective ways. Moreover, it is also thus more likely to scientifically quantify such technology; and, this has become a development trend of the total life-cycle SHM technology in modern times [4,5,6,7,8]. A series of theoretical and experimental investigations has been performed on structural damage identification, working status of structures, positions and degrees of structural damages, health monitoring sensor systems, large-scale monitoring data analysis techniques and computer vision measurement, etc., by scholars. As for relevant research findings, they have been applied in various engineering sectors, such as speckle photography [9,10,11,12,13] global positioning systems (GPS) [13], and laser doppler vibrometers [12,13]. However, the high costs of these non-contact systems prevent their wider application. Owing to the wide availability of affordable high-quality digital imaging sensors and high-performance computers, cheaper cameras with high resolutions have found growing applications in several areas [12,13,14,15,16,17]. With these devices [5,6,7,8,9,10,11], what you see is what you get, and movement information can be shown visually. On the basis of target points or physical feature points, a discrete structural geometry morphology feature point set has been constructed in most of the existing research by extracting displacement information of a single feature point on the surface of a structure. No correlation is formed between points. On the contrary, a holographic visual sensor is utilized in this paper to acquire structural geometric morphology and then build a set of feature points by virtue of full-field structural geometry monitoring. In this way, full-field measurement can be fulfilled. Compared with another method of using a discrete feature point set to construct spatial geometric morphology, a set of feature points with strongly correlated information is established on the basis of geometry morphology. Therefore, physical continuity conditions and spatial contact information between points can be sufficiently reserved. Thus, the real change in structural properties can be accurately reflected under various damage/action conditions. To summarize, contact health monitoring sensors or non-contact sensors are primarily used in the existing literature about SHM theories and methods to identify static or dynamic characteristic parameters of structures; and, in combination with finite element analysis (FEA) and experimental analysis, structural damage identification and working status estimation are performed based on measured responses of the structure, so as to reveal whole-process static/dynamic behavior, mechanisms and evolutionary rules of the structure under actions of complex loads and environmental coupling. Thanks to definite physical meanings, these approaches have been employed in practical projects [18,19,20,21,22,23,24,25,26,27,28,29]. However, they remain very vulnerable to limitations of corresponding technical conditions. Due to difficulties in conducting anomaly detection of massive monitoring data, multiple degrees of freedom in structures, strong correlations among components and a complicated relation between global and local attributes of the structure, not only is it less likely to truly reflect structural damage characteristics by a small number of sensors, but a difficult also lies in accurately presenting global or local damages by virtue of the overall structural response. Moreover, in terms of errors, both big data analysis technology and damage identification factors should be featured with certain robustness and sensitivity during the relevant test that is subjected to noise interference and errors generated by signal transmission and analysis. In other words, they should be able to identify global damages by truthfully revealing global attributes of the structural behavior and also possess high sensitivity in local damage identification.

Regarding structural geometric deformation monitoring, it is both a key component of health monitoring in the field of bridge engineering [20,21,22,23,24,25,26,27,28,29] and a critical index of structural behavior evaluation for bridges. To a certain extent, anomalous changes in structural geometry give embodiments to present safety status of bridges in a real sense; and, different degrees of structural damages or defects in long-term service time are also embodied in such anomalous changes. As for structural geometric deformation monitoring of long-span bridges [23,24,25,26,27,28,29] and in order to monitor mechanical parameters of and environmental effects on bridges, a limited number of contact sensors should be arranged on key monitoring points of the bridge structure to form a sensor array on one hand; and, on the other hand, a health monitoring system should be established by integrating them with other monitoring sensors. Alternatively, manual inspections are regularly carried out for control points of the bridge area monitoring by a total station, a level gauge and a theodolite. Unfortunately, the utilized sensors should be calibrated on a regular basis and control points for monitoring are also restricted by topographic conditions and types of the bridge structure. Consequently, these SHM approaches with poor economic efficiency only produce a limited number of structural geometric deformations on discrete monitoring points. It is much less likely to acquire holography geometric morphology which truthfully represents global and local safety status of the bridge structure.

On account of the above research, philosophy of the full-field structural geometry monitoring is combined with structural holographic technology. Structural holographic technology is derived from holography and front-projected holographic displays. Based on computer-vision-based measurement technology and non-contact geometric morphology monitoring technology, a visual sensor is comprehensively adopted to acquire original segmental dynamic and static video monitoring data to the largest possible extent. Such data contain all the structural information (i.e., geometric parameters, mechanical behavior responses, structural performance parameters, structural state parameters, load effects, and environmental activity) that is available to digital twins. In these data, information (i.e., optical information and phase information) of various points on the structural surface are truthfully and accurately recorded. In principle, three-dimensional space images of the original structure can be dynamically reproduced without mutual interference. Hence, dynamic interaction between the real structure and the virtual digital model is realized. In this way, a holographic visual sensor is proposed based on morphological monitoring data and series data of holographic images. Moreover, these data and images are generated by laboratory loading tests on a scale model of a super-span self-anchored suspension (SAS) bridge with a multi-damage working condition. Then, the holographic visual sensor is adopted to carry out holography geometric morphology monitoring for the bridge structure, which truthfully reflects actual structural deformations under the load. In comparison with a contact sensor-based health monitoring system, the non-contact holographic visual sensor has the potential to acquire structural holographic deformations which are more sensitive to local damages. Furthermore, the proposed method also overcomes the problem of response data discretization on observation points caused by restrictions over the quantity of sensors. Besides, information about global and local damages of the structure can be presented truthfully and continuously by the proposed holographic visual sensor.

This study aims at the lack of sufficient data supporting structure health monitoring, structural damage identification and digital twins for large-scale structures, which is a general issue in traditional single-point measurement method. A novel holographic visual sensor and algorithms have been proposed for full-field non-contact displacement and vibration measurement based on computer-vision technology, with improved efficiency and reduced cost. The sensor and algorithms can be applied in the full-field geometry monitoring of engineering structures. The paper is organized as follows: Section 2 covers the theoretical background of holographic visual sensor, and the holographic geometric morphology tracking algorithm is proposed, including the feature points set of structural holography geometry and the holography feature contours. Section 3 analysis with the theoretical model, and validates the proposed sensor and algorithms in full-field non-contact displacement and vibration measurement. All of the results are summarized in Section 4.

## 2. Theoretical Fundamentals

### 2.1. Holographic Visual Sensor

**Figure 1:**
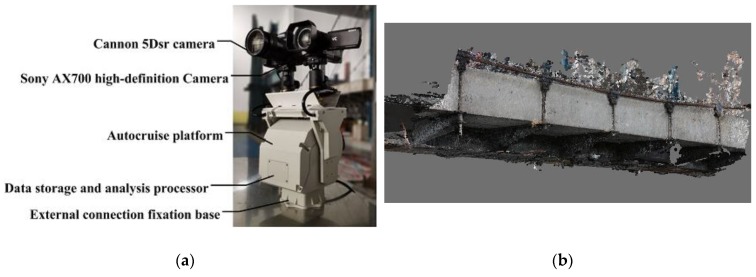
Holographic visual sensor: (**a**) main components of sensor; (**b**) holography geometric morphology of the structure.

Based on modern panoramic vision sensor technology, pattern recognition and computer technology, the holographic visual sensor is composed of an active visual sensor, an Autocruise remote control platform, an environmental monitoring element and a signal transmission and communication unit. The active observation and measurement of the structure to be measured is fulfilled by an active vision sensor (Canon 5Dsr camera and Sony AX700 high-definition camera) at the front end. Environmental monitoring units refer to temperature and humidity sensors independent of the active vision sensor. These units are used to collect environmental activity-related information of the present structure to be measured. By contrast to conventional discrete-point displacement measurement, the holographic visual sensor has the capability to arrange dense and continuous pixel-level/subpixel-level virtual measuring points on the surface of the tested object space; hence, the actual physical characteristics of structural geometry and deformation can be obtained on the whole, as shown in Figure 1.

**Figure 2:**
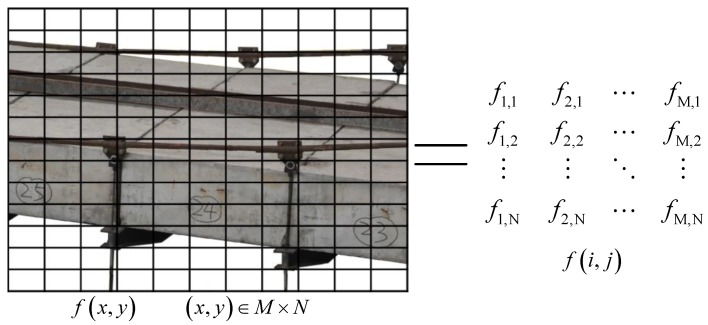
Matrix representation of pattern information digitalization.

During holography geometric morphology monitoring for bridges, autocruise parameters (i.e., preset position, on-watch position, cruise time and sampling time) are set on a computer to realize remote control over the active visual sensor, and the environmental monitoring sensor on site and acquire dynamic and static image monitoring data of the bridge structure within the present field of vision in a real-time way. In terms of full-field structural geometry monitoring, virtual measuring point positions and color information are primarily selected as measurement features for digital transformation of corresponding dynamic and static images, as presented in Figure 2. As for a static image sequence or a single-frame dynamic image sequence, the original image function *f*(*x*,*y*) is spatially discretely divided into several information zones denoted by *f*(*i*,*j*) (*i*,*j*=1, 2, …, *N*) in square meshes, as expressed in Equation (1). Here, *f*(*i*,*j*) contains RGB information, illumination information and spatial position information of the corresponding information zone. It truthfully represents all the feature information of the original image function; subsequently, spatial registration of series data, holographic feature probability edge detection and power spectral density analysis can be conducted on the basis of the feature information.

(1) $$f\left(x,y\right)=g\left(x,y\right)\cdot h\left(x,y\right)\cdot Q_{L}$$

where, *x* and *y* refers to planar coordinates of the information zone *f*(*i*,*j*) after image function discretization; *g*(·) is an incidence function that embodies the external features of an image, such as illumination intensity and environmental factors; *h*(·) represents a reflection function, standing for reflection characteristics on the structural surface and internal characteristics of the image; and, *Q_L_* is spatial position information contained in multi-time-history, multi-angle and multi-field-of-vision series data of dynamic/static images.

Normalized cross correlation analysis [30,31] is made to figure out correlations among various information zones *f*(*i,j*) in neighboring series data before and after the structure. Furthermore, holography geometric morphology with significant differences is extracted from the structure under the load or in a state of damage. Regarding the function *f*(*x*,*y*) of an *M*x*N* large original image to be searched, the Normalized Cross Correlation coefficients can be described as Equation (2).

(2) $$\delta \left(i,j\right)=\frac{\sum_{i=1}^{M}\sum_{j=1}^{N}\left|f^{x,y}\left(i,j\right)-E\left(S^{i,j}\right)\right|\cdot \left|f^{T}\left(i,j\right)-E\left(f^{T}\left(i,j\right)\right)\right|}{\sqrt{\sum_{i=1}^{M}\sum_{j=1}^{N}{\left[f^{x,y}\left(i,j\right)-E\left(S^{i,j}\right)\right]}^{2}\cdot \sum_{i=1}^{M}\sum_{j=1}^{N}{\left[f^{T}\left(i,j\right)-E\left(f^{T}\left(i,j\right)\right)\right]}^{2}}}$$

where, δ(i,j) is the Normalized Cross Correlation coefficients, $f^{x,y}\left(i,j\right)$ is the source image, $f^{T}\left(i,j\right)$ is the template, $S^{i,j}$ is the region under the template, $E\left(f^{T}\left(i,j\right)\right)$ is the mean of the template, $E\left(S^{i,j}\right)$ is the mean of in the region under the template, (*i*,*j*) represents coordinates of the original image function *f*(*x*,*y*) that denotes the information zone *f*(*i*,*j*).

**Figure 3:**
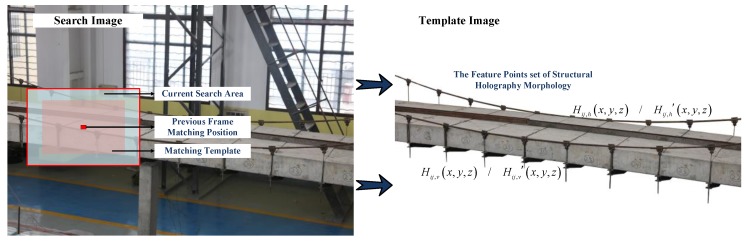
Matching schematic diagram.

As shown in Equation (2), a correlation matrix is calculated by shifting a given template *f^T^* pixel-by-pixel across a source image *f^x,y^*, which provides the information on the degree of matching between the template and the image. The maximum absolute value of the correlation matrix, whose location describes the best matching of the template, is then sought. Figure 3 is a matching schematic diagram between search image and template image; and, the region with significant differences that is figured out by Normalized Cross Correlation analysis is where holography geometric morphology of the structure changes.

**Figure 4:**
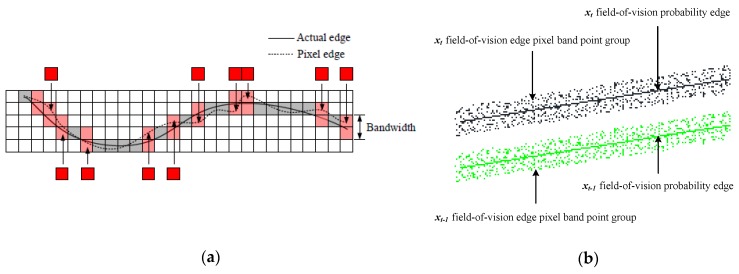
Schematic diagrams of edge pixel band point groups and probability edges: (**a**) pixel-level edges; (**b**) holographic probability edges.

As can be observed from Figure 4, transition of RGB values and gray-scale values can be found among edge contour lines extracted based on Normalized Cross Correlation computational analysis. Consequently, edges of the structure fail to be a real contour line, but an edge pixel band point group.

To quantify the acquired characteristic parameters of holography geometric morphology, a fuzzy probability-based edge detection algorithm [32,33] is adopted to extract probability edges of the structure in a complex illumination condition. Moreover, the probability edge can reflect holography geometric morphology of the structure to a significant degree. Therefore, it can be used to fulfill the experimental analysis in this study on the premise of satisfying measuring accuracy requirements. Through spatial and temporal differential analysis on holographic probability edges of the pixel band point group configuration [5], displacement of the holography geometric morphology function in horizontal and vertical directions can be expressed in the following governing Equations (3) and (4).

(3) $$\Delta_{x}=\sum_{i=1}^{m}\sum_{j=1}^{n}\left|H_{ij,h}\left(x,y,z\right)-H_{ij,h}{}^{\prime }\left(x,y,z\right)\right|$$

(4) $$\Delta_{y}=\sum_{i=1}^{m}\sum_{j=1}^{n}\left|H_{ij,v}\left(x,y,z\right)-H_{ij,v}{}^{\prime }\left(x,y,z\right)\right|$$

where, *m* and *n* represent space serial numbers of the structure’s holographic probability edge feature points; *x*, *y* and *z* are space coordinates of feature points; *H_ij_*(*x*,*y*,*z*) refers to the measured horizontal/vertical structural responses in the holography geometric morphology in a field of vision at a moment/period; and, *H_ij_’*(*x*,*y*,*z*) stands for the measured horizontal/vertical structural responses of the holography geometric morphology function in a normal state.

The *x* and *y* coordinates of these locations represent the movements in the horizontal and vertical directions. Notably, the movements obtained by applying this algorithm actually represent pixels. The real displacement can be obtained when the distance a pixel represents is known [33]. As shown in Equations (5), characteristic parameters of the holography geometric morphology are aligned between pixel and world coordinate systems on an image plane, so that degrees of freedom (DoF) of diverse pixels in the structure’s holography geometric morphology can be acquired along axes *x* and *y*, and accordingly converted into actual displacement of the structure in the world coordinate system.

(5) $$s\left[\begin{array}{c} u\\ v\\ 1 \end{array}\right]=\left[\begin{array}{ccc} \frac{1}{dX_{c}} & 0 & u_{0}\\ 0 & \frac{1}{dY_{c}} & v_{0}\\ 0 & 0 & 1 \end{array}\right]\left[\begin{array}{c} \begin{array}{cccc} f_{c} & 0 & 0 & 0 \end{array}\\ \begin{array}{cccc} 0 & f_{c} & 0 & 0 \end{array}\\ \begin{array}{cccc} 0 & 0 & 1 & 0 \end{array} \end{array}\right]\left[\begin{array}{cc} R & T\\ 0 & 1 \end{array}\right]\left[\begin{array}{c} X_{w}\\ Y_{w}\\ Z_{w}\\ 1 \end{array}\right]=\left[\begin{array}{c} \begin{array}{cccc} \alpha_{x} & 0 & u_{0} & 0 \end{array}\\ \begin{array}{cccc} 0 & \alpha_{y} & v_{0} & 0 \end{array}\\ \begin{array}{cccc} 0 & 0 & 1 & 0 \end{array} \end{array}\right]\left[\begin{array}{cc} R & T\\ 0 & 1 \end{array}\right]\left[\begin{array}{c} X_{w}\\ Y_{w}\\ Z_{w}\\ 1 \end{array}\right]$$

where, *s* is a scale factor of image projection plane transformation; *u*_0_ and *v*_0_ are base points of the pixel coordinate system; *u* and *v* represent a pixel coordinate system of the image projection plane; *X_c_* and *Y_c_* stand for a camera coordinate system; and, *f_c_* refers to an effective focal length, ***R*** to a 3 × 3 rotation matrix, and ***T*** to a translation matrix. Moreover, *X_w_*, *Y_w_* and *Z_w_* form a world coordinate system. Finally, $\alpha_{x}=f_{c}/dX_{c}$ and $\alpha_{y}=f_{c}/dY_{c}$ are both scale factors of *u* and *v*. Prior to holographic testing, all internal and external parameters can be obtained by calibrating a standard chessboard with black and white alternating [34,35,36].

Based on a feature space point set of structural holography geometric morphology and time series information of structural displacement, modal features of the structure can be further analyzed to simplify the trial bridge into a particle system connected with springs and damps. In this case, its differential balance kinetic equation can be written as Equation (6):

(6) $$M\ddot{u}\left(t\right)+C\dot{u}\left(t\right)+Ku\left(t\right)=0$$

where, $\ddot{u}\left(t\right)$, $\dot{u}\left(t\right)$ and u(t), respectively, represent an acceleration vector, a velocity vector and a displacement vector of various space points in the feature point set of structural holography geometric morphology; and, M, C and K stand for a mass matrix, a viscous damping matrix and a stiffness matrix respectively. According to Rayleigh’s hypothesis about damping [37], the damping matrix of the structure is a combination of its mass matrix and stiffness matrix, that is, C=αM+βK. Considering $K\phi_{i}=\omega_{i}^{2}M\phi_{i}$, a modal matrix Φ formed by $\phi_{1},\phi_{2}\ldots \phi_{i}$ can be solved, as shown in Equation (7) which diagonalizes the mass and stiffness matrices into modal masses $m_{i}$ and $k_{i}$ modal stiffnesses. As for geometric displacement information obtained by holography geometric morphology monitoring for the bridge structure, relevant geometric displacement coordinates can be transformed, by means of mode shape coordinate transformation, into generalized coordinates that are expressed in mode shapes. In a generalized coordinate system, an uncoupled single DoF Equation (10) can be generated according to orthogonality of displacement mode shapes. In the course of actual testing, the holography geometric morphology data are displacement sequences that contain *S* continuous time-domain signal with an equal step length. If *T* is taken to represent sampling frequency of the holographic visual sensor, then, *t*=*ST*.

(7) $$\Phi =\left[\begin{array}{cccc} \phi_{1} & \phi_{2} & \cdots & \phi_{i} \end{array}\right]$$

(8) $$\Phi^{T}Μ\Phi =diag\left(m_{i}\right)$$

(9) $$\Phi^{T}Κ\Phi =diag\left(k_{i}\right)$$

(10) $$u\left[\left(X_{w},Y_{w},Z_{w}\right),ST\right]=u\left(t\right)=\sum_{i=1}^{n}\phi_{i}\left(X_{w},Y_{w},Z_{w}\right)q\left(ST\right)$$

The matrix Φ defines modal coordinates q(t) where u(t)=Φq(t). In these modal coordinates, the equations of motion are decoupled into single degree of freedom systems defined by modal masses $m_{i}$ and modal stiffnesses $k_{i}$, and damping $c_{i}=\alpha m_{i}+\beta k_{i}$, giving the decoupled equation of motion for each mode in Equation (11).

(11) $$\ddot{q}\left(t\right)+2\xi_{i}\omega_{i}\dot{q}\left(t\right)+\omega^{2}q\left(t\right)=0$$

where the undamped natural frequency is $\omega_{i}=\sqrt{\frac{k_{i}}{m_{i}}}$, the modal damping factor is shown in Equation (12).

(12) $$\xi_{i}=\frac{c_{i}}{2m_{i}\omega_{i}}=\frac{1}{2}\left(\frac{\alpha }{\omega_{i}}+\beta \omega_{i}\right)$$

In unit impulse, *i*th mode of the structure can be figured out by solving the Equation (11).

(13) $$h_{i}\left(t\right)=\left(\frac{e^{-\xi_{i}\omega_{i}t}}{m_{i}\omega_{di}}\right)sin\left(\omega_{di}t\right)$$

where the damped natural frequency is $\omega_{di}=\omega_{i}\sqrt{1-\xi_{i}^{2}}$. Through Fourier transform of Equation (13), a complex frequency response function of the structure is acquired in a condition of arbitrary impulse, as shown in Equation (14).

(14) $$H_{i}\left(\omega \right)=\left(\frac{1}{m_{i}\omega_{di}}\frac{\xi_{i}\omega_{i}}{\xi_{i}^{2}\omega_{i}^{2}+\omega^{2}}\right)*\left(\frac{\delta \left(\omega -\omega_{di}\right)-\delta \left(\omega +\omega_{di}\right)}{i}\right)$$

Based on Equations (13) and (14), displacement response $u_{p}\left(t\right)$ of the single DoF system can be expressed in Equation (15):

(15) $$u_{p}\left(t\right)=u_{p}\left[\left(X_{w},Y_{w},Z_{w}\right),ST\right]=\sum_{i=1}^{n}A_{i}h_{i}\left(t\right)\phi_{i}\left(p\right)$$

Equation (16) gives expression to a dynamic response function of the structure:

(16) $$U_{p}\left(\omega \right)=U_{p}\left[\left(X_{w},Y_{w},Z_{w}\right),\omega \right]=\sum_{i=1}^{n}A_{i}H_{i}\left(\omega \right)\phi_{i}\left(p\right)$$

where $A_{i}$ is the amplitude of the impulse at the mode $\phi_{i}$, $\phi_{i}\left(p\right)$ is the mode shape coefficient of the degree of freedom p of the object for mode i. Here, a feature space point set of structural holography geometric morphology and the time series information of structural displacement are acquired by a holographic visual sensor. On this basis, spatial and temporal series data are analyzed to fulfill holography geometric morphology monitoring and analyze modal features. In the process of analyzing, each point in the space point set is constantly invoked to compute and analyze the corresponding local geometric displacement information; in this way, a motion-associated signal spectrum, mode shapes denoted by $\phi_{i}$ and frequency $\omega_{i}$, all required by the overall dynamic response parameter, can be established. In test conditions of this study, test objects and material behavior remain basically unchanged; by contrast, structural stiffness may alter in a working condition of being damaged. In this context, the holographic visual sensor is utilized to monitor structural holography characteristic variations. Therefore, a foundation can be laid for subsequent further research on structural behavior evolution and intelligent damage identification based on holographic spatial and temporal series data.

### 2.2. The Spatial and Temporal Series Data

**Figure 5:**
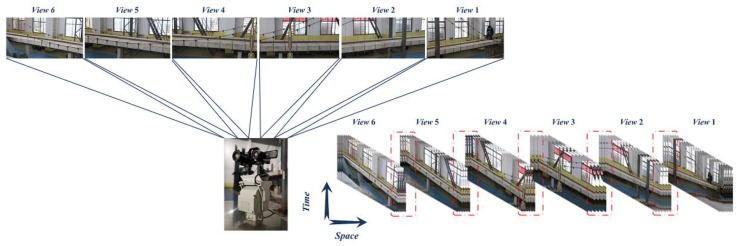
The measured spatial and temporal series data.

Raw data acquired by a holographic visual sensor have the following characteristics. First is multi-time-history. Different holography geometric data that are collected in different fields of vision have different time history in the entire testing process. The second characteristic is multi-field-of-vision. As far as technical and economic analyses are concerned, local holography geometric morphology takes form in different fields of vision by utilizing a small number of devices to perform geometric monitoring on structural local details in corresponding fields of vision. The third characteristic is known as being multi-angle. When data acquisition is implemented at on-watch positions by the Autocruise remote control platform, equivalent transformation of various angles should be performed during holography geometric data processing. The last characteristic is embodied in a strong correlation between time and space. In terms of time history and space information, the raw data are under random effects of the entire bridge structure at the current moment or period; and, structural responses in a local field of vision reflect the total structural behavior to diverse degrees. On this basis, the spatial and temporal series data that are based on the holographic visual sensor are listed in Figure 5.

Targeted at characteristics of a series data pool, time and space pointers are established for spatial and temporal series data by virtue of Matlab cell array principles. To be specific, the time pointer (i.e., time dimension) serves as an information label of the current damaging working condition and field of vision sequence; and, the space pointer, or space dimension, is deemed as an associated information label of the current local structural region relative to the overall structural position. Respectively, labels of time-history information, space field of vision, angle information and environmental information are established for raw data to carry out data integration and storage. Hence, preparations are made for further processing and analysis of subsequent data. In this way, label information of corresponding elements in the Matlab cell array can be expressed in Equation (17) below.

***Q*** {*i*, *j*}(*m*, *n*)={ ***Q***_*Time*_, ***Q**_Space_*, ***Q**_Angle_*, ***Q**_EnvironmentalAction_*, ***Q**_GrayValue_* } (17)

where, ***Q*** represents a Matlab cell array for multi-time-history, multi-angle and multi-field-of-vision series data of dynamic/static images; *i* is the serial number of damages working conditions of the test bridge and it ranges from 1 to 12 in this experiment; *j* refers to label position information in different damage working conditions, where 1 stands for *Time*, 2 for *Space* and 3 for *Angle* ...; *m* refers to a parameter to invoke label information data values of ***Q****_Time_*, ***Q****_Space_*, ***Q****_Angle_*, ***Q****_EnvironmentalAction_*, and ***Q****_GrayValue_* in a local field of vision; *n* is also the serial number of repeated measurements in the same damage working condition, or can be referred to as time information of multiple measurements; ***Q****_Time_*, ***Q****_Space_*, ***Q****_Angle_* and ***Q****_EnvironmentalAction_* are information matrices of time, space, angle and environmental labels; and, ***Q****_GrayValue_* stands for RGB values and gray values of a dynamic/static image measured by the holography geometric morphology monitoring system in a local field of vision.

**Figure 6:**
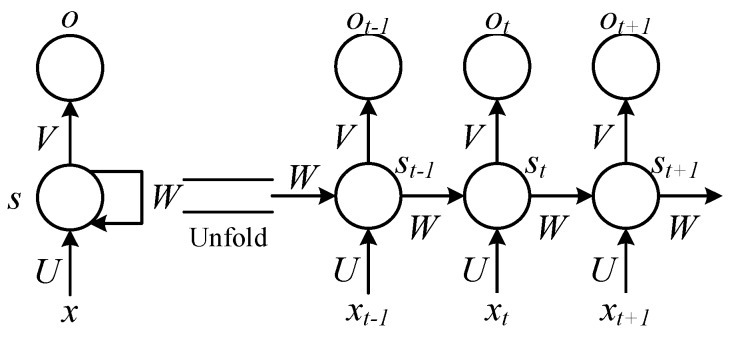
Local network structure of intelligent perception.

To process dynamic/static image monitoring series data with strongly correlated spatial and temporal information, a multi-task multi-agent action network is built based on spatial and temporal series data. According to a schematic diagram of the local network structure (see Figure 6), holography geometric morphology of the entire bridge structure can be intelligently perceived. In time dimension, information of holography geometric morphology is extracted in the same field of vision, while overall holography geometric characteristics of the structure can be extracted from diverse fields of vision in a space dimension.

where, *x_t_* is input at time *t*, ***X****=*[*x*_1_,...,*x_t_*_−1_,*x_t_*_+1_,...*x_T_*] is an input sequence; *s_t_* is a hidden state at time *t*, multiple neurons are included and ***S****=*[*s_1_,...,s_t-1_,s_t+1_,...s_T_*]; *h* denotes output at time *t*, ***H****=*[*h*_1_,...,*h_t_*_−1_,*h_t_*_+1_,...*h_T_*] is an output sequence; and, ***U*** stands for a weight parameter matrix of the input sequence ***X***, ***W*** for a weight parameter matrix of the hidden state ***S***, and ***V*** for a weight parameter matrix of the output sequence ***H***. In this case, if a mathematical model of the current hidden state *s_t_* can be expressed as *s_t_*=*F*(*Ws_t_*_-1_,*Ux_t_*), where *F*(·) stands for an activation function of the hidden state, then, the mathematical model of output is *h_t_*=*G*(*Vs_t_*) (*G*(·) as an activation function of output).

## 3. Experimental Study

### 3.1. Experimental Setup and Procedure

**Figure 7:**
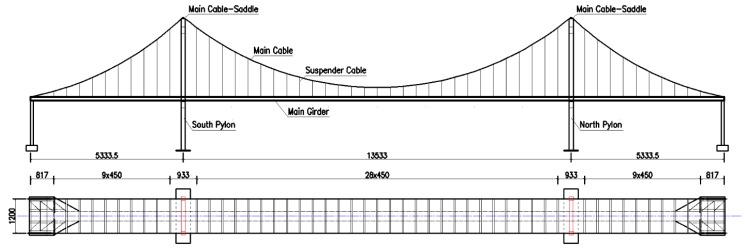
The construction diagram of test bridge (total length of bridge is 24 m, Units: mm).

With the help of the existing deep machine vision-based measuring technique available to the research group [5,38], the holographic visual sensor is selected as the experimental facility to test structural mechanical behavior of a scale test model of a super-span SAS bridge in the whole process of variable load conditions and holography geometric morphology. In this way, full-field non-contact measurement is investigated for holography geometric morphology of the test bridge in various working conditions under complex uncertainties. Besides, a data pool can be also constructed for whole-process holography geometric morphology monitoring information of the test model. A conventional contact high-precision geometric deformation measuring instrument (i.e., dial gauge and accelerometer) is utilized to validate the measured data of test model deformation in corresponding working conditions and improve the proposed holography geometric morphology monitoring approach. Considering that loading tests under variable loads should be carried out for the test model in different working conditions, structural damages are rather sensitive to geometric morphology changes. Therefore, the scale test model of super-span SAS bridge with a total longitudinal length of 24 m (see Figure 7) is selected as the test object here, because its damaged structural members can be easily repaired, the structure itself has abundant feature points and its performance and mechanical behavior [39,40,41,42,43,44] have been also explored by the research group at the earlier phase.

**Figure 8:**
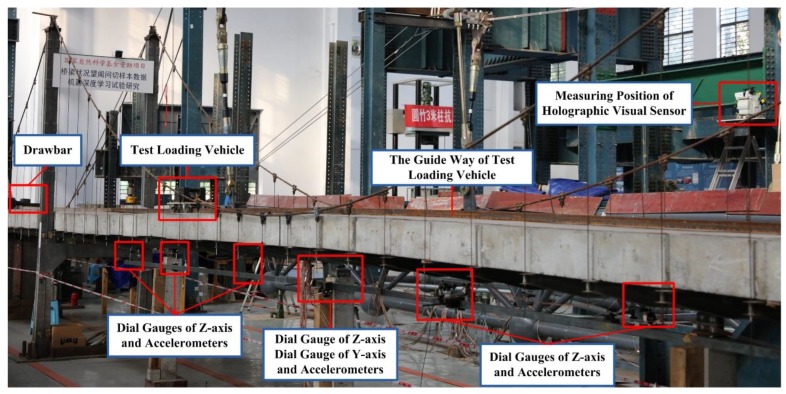
Experiment site layout.

As presented in Figure 8, an experimental holographic visual sensor is used to fulfill the site layout for holography geometric morphology monitoring of the test bridge. Known controlled variables of the experiment include environmental effects, vehicle load conditions and structural behavior. Subjected to diverse test working conditions in which the holographic visual sensor is used to monitor holographic morphology of the test bridge, ambient parameters of the laboratory, such as temperature, illuminance and humidity, all exert certain influence on test results. For the purpose of maintaining comparatively stable raw series data under diverse loads, a test measurement was conducted in comparatively constant and ideal indoor environmental conditions at 9:00 A.M. from July to October 2019. To be specific, these environmental conditions include good illuminance, an average temperature at about 28 °C and an average humidity of 70% approximately. For practical use, the measurement may be conducted under circumstances in which illuminance, temperature, and humidity are comparatively stable in different seasons, time, and weather conditions. Moreover, these conditions are used as label information of the environment, to help note down relevant parameters.

**Figure 9:**
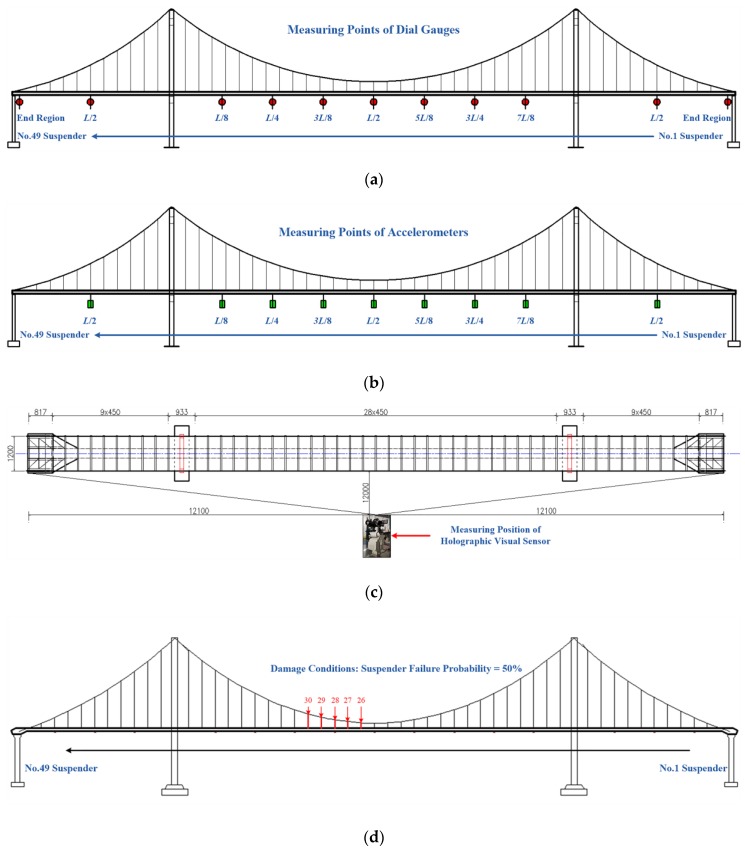
Sensor layout: (**a**) measuring points of dial gauges; (**b**) measuring points of accelerometers; (**c**) measuring position of holographic visual sensor (units: mm); (**d**) manual damages.

The test scheme is primarily divided into 2 parts. As for the arrangement of measuring points, it is presented in Figure 9 below.

**Figure 10:**
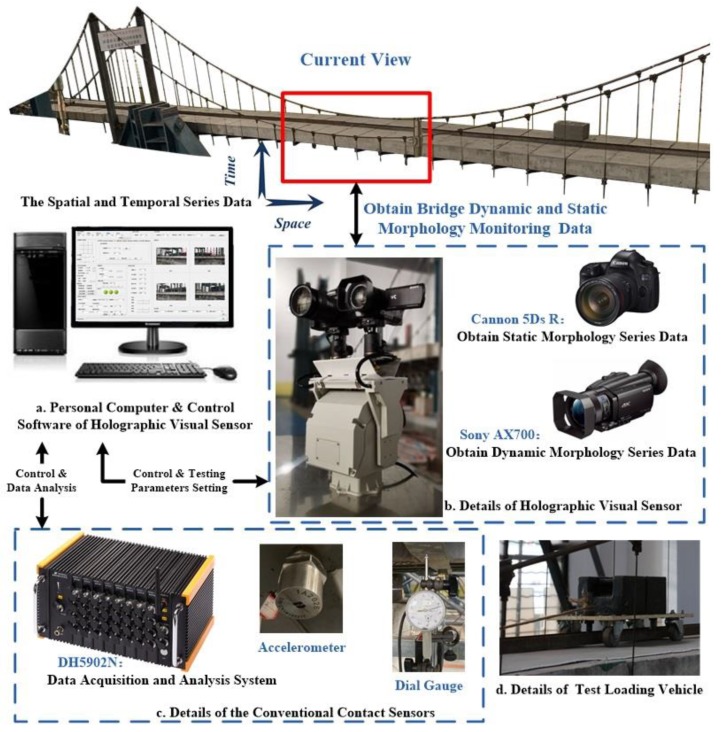
The device of experiments.

The first part is a single-point excitation. An impact hammer was used to complete single-point excitation for the test bridge with different impact forces, monitor automatic cruise situations of the system, continuously collect holography geometric morphologies in diverse working conditions of the test scheme in a segmented manner, and acquire holographic deformation and dynamic response curves in the respective working conditions of the test bridge. The second part of this scheme is running vehicle excitation. A test loading vehicle with a load of 25 kg/50 kg/100 kg moves on the bridge at 0.5 m/s, in which case, the monitoring system begins to cruise automatically and continuously acquire holography geometric morphologies in various test conditions; thus, holographic deformation and dynamic response curves of the test bridge are generated for different working conditions. According to this scheme, two measurement modes were adopted. One is traditional, and the other is holographic visual measuring. As for the former, the midspan measuring points of the test bridge include the *L*/8, *L*/4, 3*L*/8, *L*/2, 5*L*/8, 3*L*/4 and 7*L*/8 key cross-sections, while those of its side span only consist of the *L*/2 key cross-section; in addition, a displacement sensor and an accelerometer are respectively provided on the abutments at both ends to acquire structural responses. As far as the latter is concerned, a holographic visual sensor is used as an experimental facility to fulfill measurement, as shown in Figure 10.

Test content: Subjected to different modes of excitation and manual damages on different positions of the sling, holography geometric morphology monitoring series data of dynamic/static images are firstly tested for the test bridge. Secondly, in different conditions, measured deformations on measuring points are tested by a conventional contact displacement sensor and an accelerometer in corresponding working conditions. As for the test conditions and content of this paper, they have been presented in Table 1. Regarding manual damages, beam anchorage device of the catenary wire is manually adjusted, and different values of the cable force represent varying damage degrees. Once the anchorage device is entirely released, the corresponding failure probability reaches 100%. Here, the failure probability is 50%, as shown in Figure 9d.

### 3.2. Holographic Visual Sensor Based Characterization Parameters of Morphology Results

**Figure 11:**
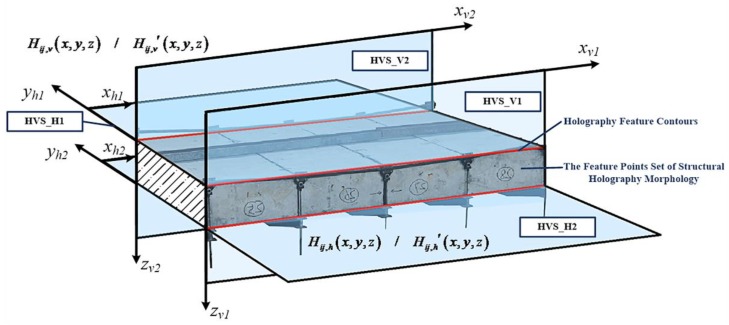
The feature points set of structural holography morphology and feature contours.

Each frame of the test bridge’s spatial and temporal series data in various test conditions were separately extracted to successively construct time-history information label, space field of vision label, angle information label and environmental information label, so that pixel resolution could be figured out. Moreover, noise signal filtration was performed by a denoising and anti-disturbance element, a Euler motion amplification element and a motion information extraction element [45]. As a result, a structural holographic morphology which is formed by feature point sets of the structural space was achieved, as shown in Figure 11. In space, all holographic feature points roughly form 4 space fitting surfaces which are denoted as HVS_H1, HVS_H2, HVS_V1 and HVS_V2. They truthfully, continuously and sensitively reflect changes in structural holography morphology in the whole process of test. By configuring space constraint conditions and physical continuity conditions at junctions of the space fitting surfaces for the test bridge in a holographic morphology [5], the holography feature contours that contain structural geometric displacement information were solved. This is deemed as a testing basis for structural morphology monitoring that is implemented by a holographic visual sensor. On account of this, pixel-level or subpixel-level virtual measuring points may be densely and continuously distributed on surface of the test object space. As a result, not only are actual geometric morphology and deformation shape characteristics of the structure acquired on the whole, but also time–history curves of structural displacement can be extracted, and the modal analysis fulfilled.

**Figure 12:**
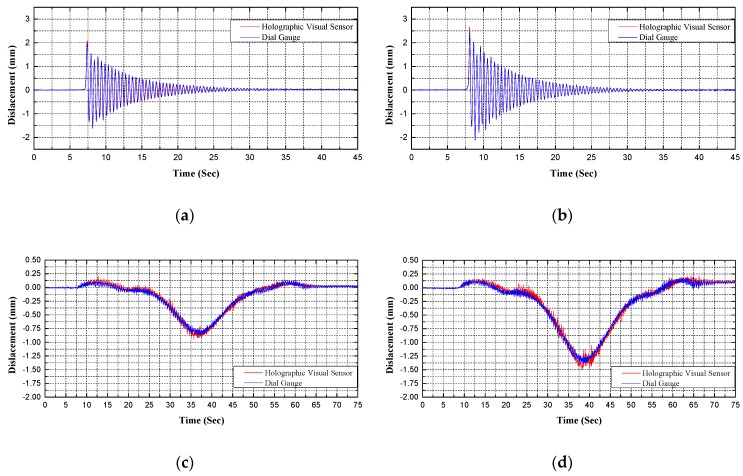
Comparison of displacement responses obtained from test results: (**a**) time history of displacement in B1 condition; (**b**) time history of displacement in B2 condition; (**c**) time history of displacement in C1 condition; (**d**) time history of displacement in C2 condition.

Using holographic visual sensor, the results of structural geometry monitoring are shown in Figure 12 and Table 2.

Through comparative analysis on Figure 12 and Table 2, the following summaries are demonstrated by test results obtained by the proposed holographic visual sensor and the conventional contact sensor. In B condition of single-point excitation and C condition of running vehicle excitation firstly, their dynamic displacement response curves present a basically identical variation tendency on the whole for the *L*/2 test cross-section in midspan of the test bridge; in other words, coincidence between them is rather high and corresponding data are stable and highly reliable. This proves accuracy and feasibility of the proposed approach. Secondly, the proposed method can be used to represent all actual dynamic displacement responses of feature point sets for the structural surface space during holographic morphology monitoring; and, it can provide more abundant, more complete and more comprehensive information about the structural mechanical performance if compared with conventional displacement sensors in discrete point arrangement. Thirdly, several points are chosen to compare, and the error rate is within 5%, RMSE is less than 0.5; clearly, such accuracy meets the demand of engineering practice.

**Figure 13:**
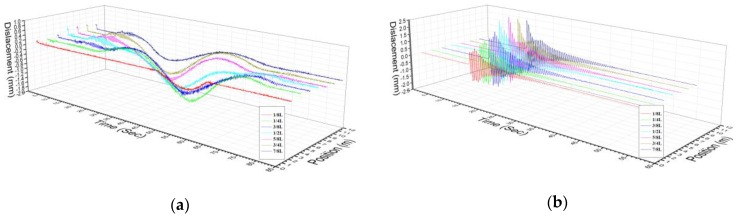
Results of all dial gauges measuring points by holographic visual sensor: (**a**) C test condition; (**b**) B test condition.

As presented in Figure 13, dynamic displacement response curves are generated from holographic morphology monitoring on the midspan of the test bridge. Good synchronization is found between these curves and data produced by 7 displacement sensors that are arranged on the corresponding test cross-section. This further signifies the superiority of the holographic visual sensor in monitoring whole performance of the bridge structure. In addition, it is also demonstrated that the proposed sensor has the capability to effectively solve the defects which are incurred from the disadvantages (e.g., limited number, limited arrangement space, regular calibration required and discrete-point measurement) of conventional measuring instruments.

**Figure 14:**
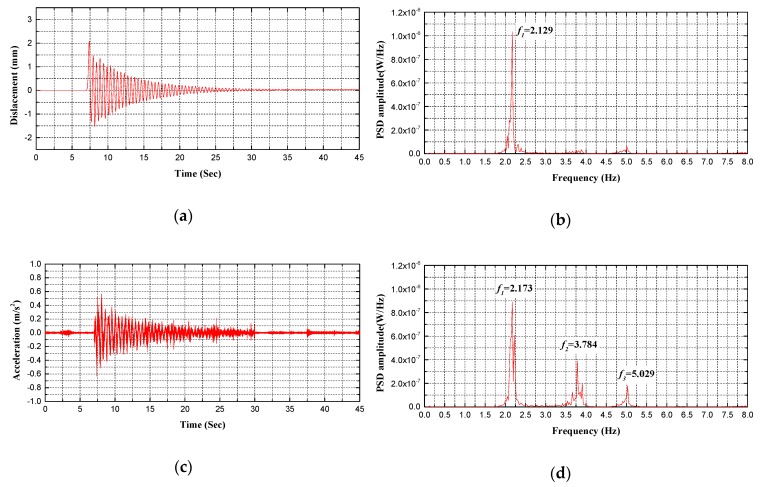
Comparison between measured frequencies for B1 test condition: (**a**) displacement response measured by holographic visual sensor (HVS); (**b**) the power spectral density (PSD) of displacement response measured by HVS; (**c**) acceleration signal measured by accelerometer; (**d**) the PSD of acceleration signal measured by accelerometer.

**Figure 15:**
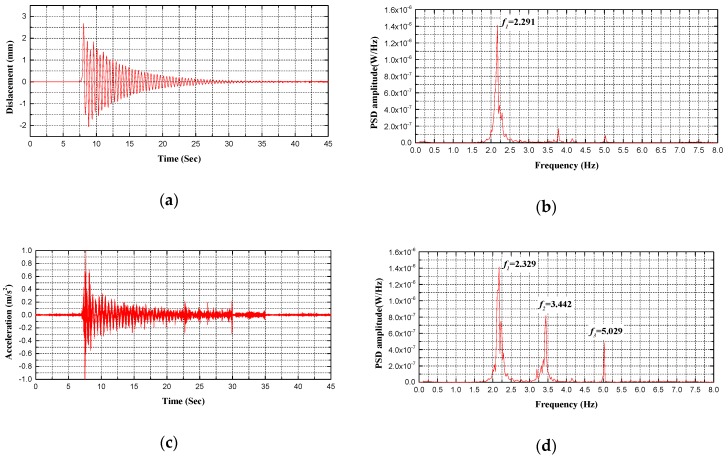
Comparison between measured frequencies for B2 test condition: (**a**) displacement response measured by HVS; (**b**) the PSD of displacement response measured by HVS; (**c**) acceleration signal measured by accelerometer; (**d**) the PSD of acceleration signal measured by accelerometer.

**Figure 16:**
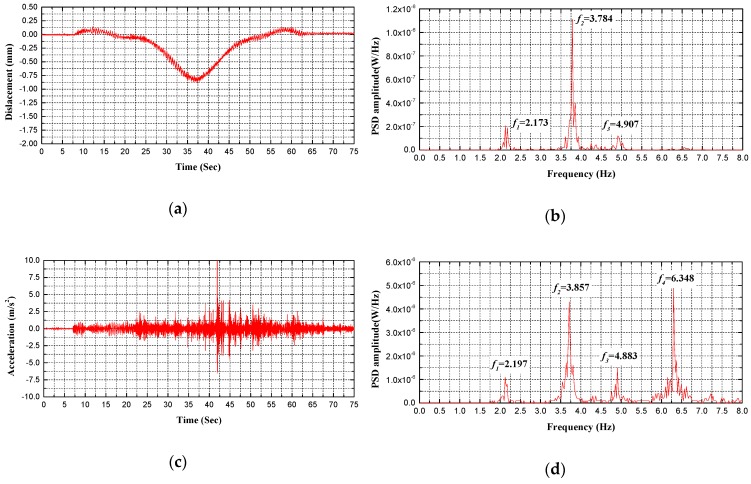
Comparison between measured frequencies for C1 test condition: (**a**) displacement response measured by HVS; (**b**) the PSD of displacement response measured by HVS; (**c**) acceleration signal measured by accelerometer; (**d**) the PSD of acceleration signal measured by accelerometer.

**Figure 17:**
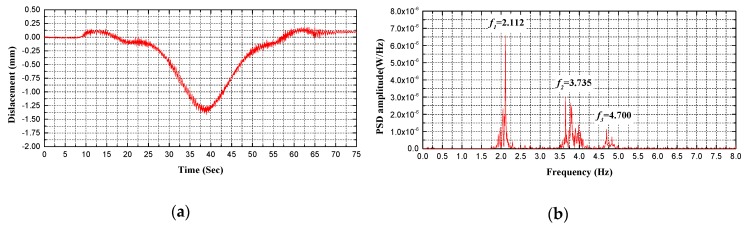

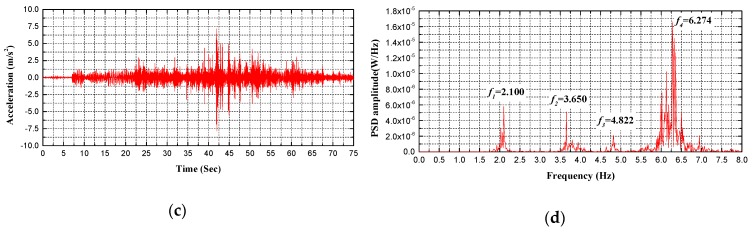
Comparison between measured frequencies for C2 test condition: (**a**) displacement response measured by HVS; (**b**) the PSD of displacement response measured by HVS; (**c**) acceleration signal measured by accelerometer; (**d**) the PSD of acceleration signal measured by accelerometer.

Regarding bridge performance and safety evaluation, the proposed holographic visual sensor together with the corresponding structural morphology monitoring method can be deemed as an efficient, continuous and convenient preliminary holographic displacement monitoring technique. Spectral analysis can be thus made on dynamic displacement signals that are acquired through holography morphology monitoring to further achieve modal parameters of the test bridge. Without a doubt, these parameters are beneficial for more comprehensive evaluation on bridge performance. As given in Figure 14, Figure 15, Figure 16 and Figure 17, specific to the *L*/2 test cross-section in midspan of test bridge, Power Spectral Density (PSD) functions are obtained in various test conditions based on holographic morphology displacement response curves, acceleration signal curves and spectral analysis on them.

In test condition B of single-point excitation (see Figure 14 and Figure 15, Table 3), the following facts have been demonstrated by holographic morphology displacement response curves, acceleration signal curves and spectral analysis on them specific to the *L*/2 test cross-section in midspan of the test bridge. First, the holographic visual sensor applied in this study is able to generate the natural frequency of the structure through testing in easier ways. Second, regarding the natural frequency of the power spectral density function between the displacement response and acceleration signal, its error rate is within 2.5% and the RMSE is less than 0.5 in the first mode. Third, the influence of environmental noise and illumination, etc., results in a certain loss of displacement response signal during the test; power spectral density function in second mode greatly differs from that in third mode; however, it is still likely to acquire natural frequencies of the test bridge in second and third modes (second mode: 3.6 Hz, third mode: 5.1 Hz) and they basically coincide with situations of the acceleration signal (second mode: 3.78 Hz, third mode: 5.03 Hz). If the influence of external factors such as environmental noise and illumination can be further eliminated, integrity of displacement response signals can be effectively enhanced so that more abundant structural performance information is retained.

In test condition C of the running vehicle excitation specific to the *L*/2 test cross-section in the midspan of the test bridge (see Figure 16 and Figure 17, Table 3), the following can be observed from the holographic morphology displacement response curves, acceleration signal curves and spectral analysis on them: First, the holographic visual sensor applied here is able to generate frequency of structure through testing in easier ways. In addition to a rather high goodness of fit, the data are stable and highly reliable, which validates the accuracy and feasibility of the proposed method. Second, the load effects of the test bridge in actual service are principally simulated in such a test condition; and, the test signal data block includes the forced vibration of the bridge. To acquire the natural frequency of the test bridge, residual vibration signals are intercepted by extending the test time when the test vehicle moves away. Through analysis, it turns out that its error rate is within 3% and the RMSE is less than 0.6. Third, under the influence of external excitation signals, the power spectral density function peaks in diverse modes are significantly different from each other; however, frequencies at all modes are accurately collected based on displacement response and acceleration signals of the holographic visual sensor. Moreover, the corresponding analytical results are in good coincidence. At last, temporal dependency is indicated by the frequency responses of the test signal. In the course of time-frequency analysis, a proper analytical method (e.g., the Fourier transform, Welch’s method) should be selected in line with signal stationarity.

The holographic visual sensor-based bridge morphology monitoring cannot be separated from an active-pixel visual sensor and the utilization of visual measurement techniques to acquire holographic morphology features. Although the instrumental errors of the active-pixel visual sensor may affect the accuracy of original static/dynamic series data to a certain extent, they can be almost completely erased by using differential analysis on the edge contour lines and morphological feature points of the bridge structure. Incidentally, the differential analysis is also applied in acquiring structural displacement information in the context of holographic morphology feature analysis. For the purpose of attaining major reasons why the edge detection algorithm that is used to collect structural holography morphology gives rise to errors incurred in the test, probability edges of the structure are constructed by processing and analyzing pixel band point groups under circumstances that, instead of acquiring actual edge contour lines of the test bridge, pixel band point groups are obtained for the structural edge in most cases. The probability edges that can be used for experimental analysis here, not only reveal structural holography morphology to a great extent, but also satisfy requirements for measurement accuracy. In spite of this, differences between probability edges and the actual edge contour lines of the structure bring about major errors of bridge morphology monitoring test. Additionally, experimental measurement results are vulnerable to noise interference of the system, drastic changes in ambient light and insufficiency of illumination intensity as experimental scenarios of this study are comparatively complicated. Consequently, edge detection operators fail to detect probability edges in the most effective ways. For this reason, the existing edge detection algorithm—that is, an obscure edge pixel point group—is replaced with an optimal edge line, and can be modified and optimized in subsequent research to reduce errors and improve measurement accuracy and algorithm stability [46,47]. Concerned with environmental interference and noise influence, noise reduction anti-disturbance autoencoders (i.e., denoising AE (DAE) and contractive AE (CAE)) are introduced into this paper with the goal of lowering relevant errors and improving measurement accuracy and algorithm stability. For random noise signals, the corresponding elimination effect is poor. Based on the assumptions of active noise reduction, an environmental noise signal acquisition sensor may be arranged at the monitoring site in practice on one hand; on the other hand, a noise database can also be constructed. In this way, random environmental noise removal efficiency is improved [5,48,49,50,51,52,53].

### 3.3. Structural Geometry Monitoring Analysis Using the Spatial and Temporal Series Data

**Figure 18:**
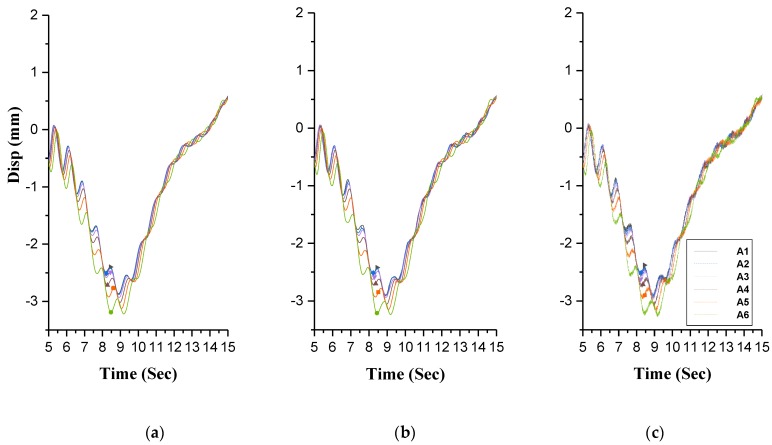
The test bridge displacement time histories of the 1/2 midspan under various damage/activities: (**a**) theoretical calculation results by Midas; (**b**) measured values based on the proposed method; (**c**) measured values based on displacement sensors.

Figure 18 presents bridge morphology monitoring test results based on the holographic visual sensor, measurement results based on conventional contact sensors, and calculation results based on the full-bridge finite element analysis by Midas. Here, only concrete situations about 1/2 of the midspan are listed for explanations.

As can be seen from Figure 18, the curve shapes and variation tendencies of testing results generated by the proposed method, by the sensors and by theoretical calculations are basically consistent with each other, respectively. The proposed method here performs well in both identification and displacement information extraction. Then, noise signals at different levels can be found in measured curves based on the displacement sensors and the proposed algorithm, due to the gray value input of ambient noise information during the laboratory test; moreover, noise signals produced by the displacement sensor are far greater than those of the proposed algorithm. In comparison with conventional contact displacement sensors, holographic visual sensor-based morphology monitoring overcomes a problem of local deformation information loss in fitted curves of limited measuring points as relevant damages cause deformation. In various damage working conditions, obvious differences in curves can be observed. This can be used as the basis for effective identification of test bridge damage degrees and positions. Finally, displacement time-history curves and maximum displacement at 1/2 midspan present different variation tendencies in respective damage/operating conditions. It manifests that holography geometric morphology monitoring on the bridge structure has the potential to reveal actual changes in structural behavior truthfully, continuously, sensitively and rather accurately. However, this study is merely aimed at a multi-damage working condition where the suspender failure probability is equal to 50%, which plays an insignificant role in structural behavior variations that are caused by structural damages of bridges in practical services. Therefore, how to quantify minor differences in curves and amplify damage features by virtue of a microscopic theory [54,55,56] for structural micro-variation characteristics should be further investigated by the research group.

## 4. Conclusions

An emerging holographic visual sensor and algorithms have been proposed for full-field non-contact displacement and vibration measurements based on computer-vision technology, with improved efficiency and reduced cost. Targeted at a test model of a super-span self-anchored suspension (SAS) bridge, holography geometric morphology monitoring tests are successively performed in multi-damage/operating conditions. The observations of this study can be summarized as follows:

(1) Laboratory experiments on 24 m-span self-anchored suspension bridge demonstrate that holographic full-field displacement and vibration signal can be accurately, sensitively and simultaneously measured in multi-damage/operating conditions using holographic visual sensor, and the identified full-field displacements and natural frequencies by the holographic visual sensor match well with those by using dial gauges and accelerometers.
(2) The holographic visual sensor can arrange dense and continuous pixel-level/subpixel-level virtual measuring points on the surface of the tested object space, and the denser full-field displacement and smoother mode shapes can be further extracted, which makes it possible to dynamically update a model of digital twins, structural condition assessment, and intelligent damage identification methods.
(3) As raised in this study, a holographic visual sensor is utilized to monitor holography geometric morphology of the bridge structure dependent on series data of structural holography images. In terms of normal deformations free of damages and abnormal deformations with damages, holography geometric morphology monitoring shows a strong capability in identifying their features, and accurately reflecting the real change in structural properties under various damage/action conditions.
(4) It is much more likely for the test results here to suffer the influence of system noise interference and dramatic ambient light changes. Moreover, different illumination intensities cause certain structural response signal differences and losses. Concerned with environmental interference and noise influence, a microscopic theory and noise reduction anti-disturbance autoencoders (i.e., denoising AE (DAE) and contractive AE (CAE)) are recommended in this study, with the goal of lowering relevant errors and improving holographic visual sensor accuracy and algorithm stability. Once actions of noise and illuminance, etc., are further eliminated, not only can the integrity of response signals be effectively improved, but more abundant structural performance information can be retained.

In summary, the holographic visual sensor shows significant potentials as a credible, high frequency, low-cost alternative to conventional displacement and acceleration sensors for SHM, especially for structures whose displacement responses are causing concerns which are difficult or expensive to obtain using conventional sensors, e.g., long-span bridges, high-rise buildings, wind turbine blades, etc.

## Figures and Tables

**Table 1:** Test conditions and content.

Test Conditions	Serial No. of Working Conditions	Test Variables		
		Test Load (kg)	Test Velocity (m/s)	Damage Conditions (Suspender Failure Probability = 50%)
**Damage Conditions**	A1	100	0.5	/
	A2	100	0.5	26
	A3	100	0.5	26 + 27
	A4	100	0.5	26 + 27 + 28
	A5	100	0.5	26 + 27 + 28 + 29
	A6	100	0.5	26 + 27 + 28 + 29 + 30
Single-Point Excitation	B1	30	/	/
	B2	60	/	/
Running Vehicle Excitation	C1	25	0.5	/
	C2	50	0.5	/

**Table 2:** Test results comparison of holographic visual sensor (HVS) and dial gauges.

Test Conditions	Test Methods	Maximum Displacement Responses at Measuring Points/mm						
		*L*/8	*L*/4	3*L*/8	*L*/2	5*L*/8	3*L*/4	7*L*/8
B1	HVS/ *R_1_*	0.41	0.51	0.66	0.90	0.70	0.53	0.43
	Dial Gauges/ *R_2_*	0.40	0.49	0.68	0.87	0.69	0.51	0.41
	Error/ |*R_1_*-*R_2_*|/*R_2_*	2.5%	4.08%	2.94%	3.45%	1.45%	3.92%	4.87%
	RMSE	0.416	0.428	0.457	0.449	0.435	0.424	0.487
B2	HVS/ *R_1_*	0.67	0.88	1.14	1.47	1.18	0.99	0.68
	Dial Gauges/ *R_2_*	0.69	0.91	1.12	1.43	1.15	0.96	0.71
	Error/ |*R_1_*-*R_2_*|/*R_2_*	2.89%	3.30%	1.79%	2.80%	2.61%	3.13%	4.22%
	RMSE	0.409	0.431	0.402	0.451	0.448	0.427	0.438
C1	HVS/ *R_1_*	0.86	1.12	1.49	2.07	1.61	1.16	0.89
	Dial Gauges/ *R_2_*	0.89	1.09	1.52	2.03	1.57	1.13	0.91
	Error/ |*R_1_*-*R_2_*|/*R_2_*	3.37%	2.75%	1.97%	1.97%	2.55%	2.65%	2.20%
	RMSE	0.429	0.447	0.435	0.461	0.463	0.436	0.447
C2	HVS/ *R_1_*	1.19	1.52	1.94	2.51	2.05	1.56	1.22
	Dial Gauges/ *R_2_*	1.23	1.49	1.97	2.46	1.99	1.52	1.25
	Error/ |*R_1_*-*R_2_*|/*R_2_*	3.25%	2.01%	1.52%	2.03%	3.01%	2.63%	2.45%
	RMSE	0.443	0.442	0.438	0.455	0.487	0.440	0.439

Notes: $RMSE\left(X,h\right)=\sqrt{\frac{1}{N}\sum_{i=1}^{N}{\left(h_{i}-x_{i}\right)}^{2}}$, where *X* stands for test values of the proposed method, *h* for measured values of other sensors, and *N* for data dimensions.

**Table 3:** Data comparison of HVS and the sensor in dynamic vibration.

Test Conditions	Sensor Type	Modal Frequency/Hz			
		1st	2nd	3rd	4th
B1	Holographic Visual Sensor	2.129	-	-	-
	Accelerometer	2.173	3.784	5.029	-
B2	Holographic Visual Sensor	2.291	-	-	-
	Accelerometer	2.329	3.442	5.029	-
C1	Holographic Visual Sensor	2.173	3.784	4.907	
	Accelerometer	2.197	3.857	4.883	6.348
C2	Holographic Visual Sensor	2.112	3.735	4.700	-
	Accelerometer	2.100	3.650	4.822	6.274
